# Regulatory network of ammonium and nitrate uptake and utilization in rice

**DOI:** 10.3389/fpls.2025.1656041

**Published:** 2025-10-20

**Authors:** Xiaoli Guo, Ju Zhang, Feilong Ma, Pengxiang Li, Yunlong Ma, Kedong Xu, Ti Liu

**Affiliations:** ^1^ Key Laboratory of Plant Genetics and Molecular Breeding, Henan Key Laboratory of Crop Molecular Breeding and Bioreactor, Henan International Joint Laboratory of Translational Biology, Zhoukou Normal University, Zhoukou, Henan, China; ^2^ College of Agronomy, Henan Agricultural University, Zhengzhou, Henan, China

**Keywords:** rice, ammonium, nitrate, transport, nitrogen use efficiency

## Abstract

Nitrogen (N) plays a crucial role in various aspects of crop growth, development, yield, and quality. It is essential for processes ranging from protein synthesis and photosynthesis to crop adaptation and stress tolerance, thereby having a profound impact on crop production. Crops primarily absorb N in the forms of ammonium (NH_4_^+^) and nitrate (NO_3_^-^), with NH_4_^+^ being the predominant form absorbed by flooded crops such as rice. This review focuses on rice and highlights recent significant advances in the mechanisms of N uptake and utilization, including the roles of NO_3_^-^ and NH_4_^+^ transporters. Key transporters such as *OsAMT1.1* and *OsNRT1.1B* play central roles in enhancing N uptake and improving N use efficiency (NUE). Furthermore, natural allelic variations in genes such as *DNR1* and *OsWRKY23* underlie the differences in NUE between *indica* and *japonica* subspecies. We also discuss the potential of multi-gene pyramiding strategies, such as *OsAMT1.2*×*OsGS1.2*×*OsAS1*, to synergistically improve NUE through coordinated regulation of N uptake, assimilation, and remobilization. Collectively, this review systematically summarizes the functions and regulatory mechanisms of key NUE-related genes in rice, providing valuable gene resources and a theoretical foundation for the molecular breeding of N-efficient rice varieties.

## 1 Introduction

Nitrogen (N) is a major limiting factor for crop growth and high grain yield, as it is a key component of numerous essential biomolecules, including nucleic acids, enzymes, amino acids, and proteins. Hence, it is often referred to as the ‘element of life’ (Crawford, 1995; Stitt, 1999). N deficiency is a key limiting factor in crop yield formation. However, excessive N fertilizer application not only increases economic costs but also causes serious environmental damage. Therefore, elucidating the genetic basis of N use efficiency (NUE) in crops and breeding improved varieties with both high yield and enhanced NUE is essential for reducing N fertilizer demand and promoting sustainable agricultural development. The mechanisms of efficient N uptake and utilization in plants involve multiple processes. Firstly, plant roots absorb various forms of N from the soil; secondly, N is transported and transformed within the plant, and finally, it is assimilated through the action of various enzymes. This constitutes a complex process regulated by multiple factors at different stages of crop growth and development. Plant roots absorb and assimilate different forms of N, including inorganic N (such as NH_4_^+^ and NO_3_^-^) and organic N (such as amino acids and peptides), through transmembrane transporters or ion channels. In aerobic soils, nitrate (NO_3_^-^) is the predominant form of inorganic N, whereas in flooded wetlands or acidic soils, ammonium (NH_4_^+^) is the main inorganic N form (Sasakawa and Yamamoto, 1978). NO_3_^-^ and NH_4_^+^ are absorbed through NO_3_^-^ transporters (NPF/NRTs) and NH_4_^+^ transporters (AMTs), respectively. The absorption of NO_3_^-^ or NH_4_^+^ by plant roots typically induces rhizosphere acidification or alkalization, thereby further affecting the bioavailability of soil N to plants. To cope with the heterogeneity and dynamic changes in NO_3_^-^ or NH_4_^+^ ion concentrations in soil solutions, plants have evolved both high-affinity transport systems (HATS) and low-affinity transport systems (LATS) for NH_4_^+^ and NO_3_^-^. These systems are distributed in different plant tissues and cooperatively regulate N uptake and distribution (Crawford, 1995; Glass et al., 1992; Crawford and Glass, 1998; Forde, 2000). In rice, the HATS for NH_4_^+^ belong to the *OsAMT1* family, while the HATS for NO_3_^-^ belong to the *OsNRT2* family and its partner proteins, the *OsNAR2* family.

In most plants, a small portion of the absorbed NO_3_^-^ is assimilated in the roots, while the majority is transported to the shoots, where it is reduced to nitrite by nitrate reductase (NR) in the cytosol. It is then transported into plastids and chloroplasts and further reduced to NH_4_^+^ by nitrite reductase (NiR) (Xu et al., 2012). NH_4_^+^ derived from NO_3_^-^ reduction or directly absorbed by *AMTs* is toxic and must be assimilated in the roots via the glutamine synthetase (GS)/glutamate synthase (GOGAT) cycle into glutamine (Gln) and glutamate (Glu), which are the core molecules in plant N metabolism. Subsequently, Glu can be converted into aspartate (Asp) via aspartate aminotransferase (AAT), and Gln can be converted into asparagine (Asn) by asparagine synthetase (AS). These four amino acids (Glu, Gln, Asp, and Asn) play crucial roles in N transport within plants, transferring N from absorption sites to tissues where it is required (Xu et al., 2012).

In recent years, NO_3_^-^ and NH_4_^+^ transporters have been identified and their functions characterized in the model crop rice. Meanwhile, the regulatory mechanisms of N uptake, transport, and assimilation have also been extensively studied. This review focuses on rice and highlights recent significant advances in the mechanisms of N uptake and utilization, including the roles of NO_3_^-^ and NH_4_^+^ transporters. In addition, the functions and regulatory mechanisms of key genes related to NUE in rice are systematically summarized, providing gene resources and theoretical foundations for the molecular improvement of N-efficient rice varieties. Finally, this article emphasizes the challenges of improving NUE and advocates an integrated research approach combining molecular mechanisms, advanced technologies, and agronomic practices. By precisely coordinating N uptake, transport, assimilation, and remobilization with rice developmental responses to N availability, it is possible to ensure efficient N use, thereby contributing to global food security and the sustainable development of agriculture.

## 2 Functions of NH_4_^+^ transporters in rice

### 2.1 Classification and transport characteristics

**Figure 1:**
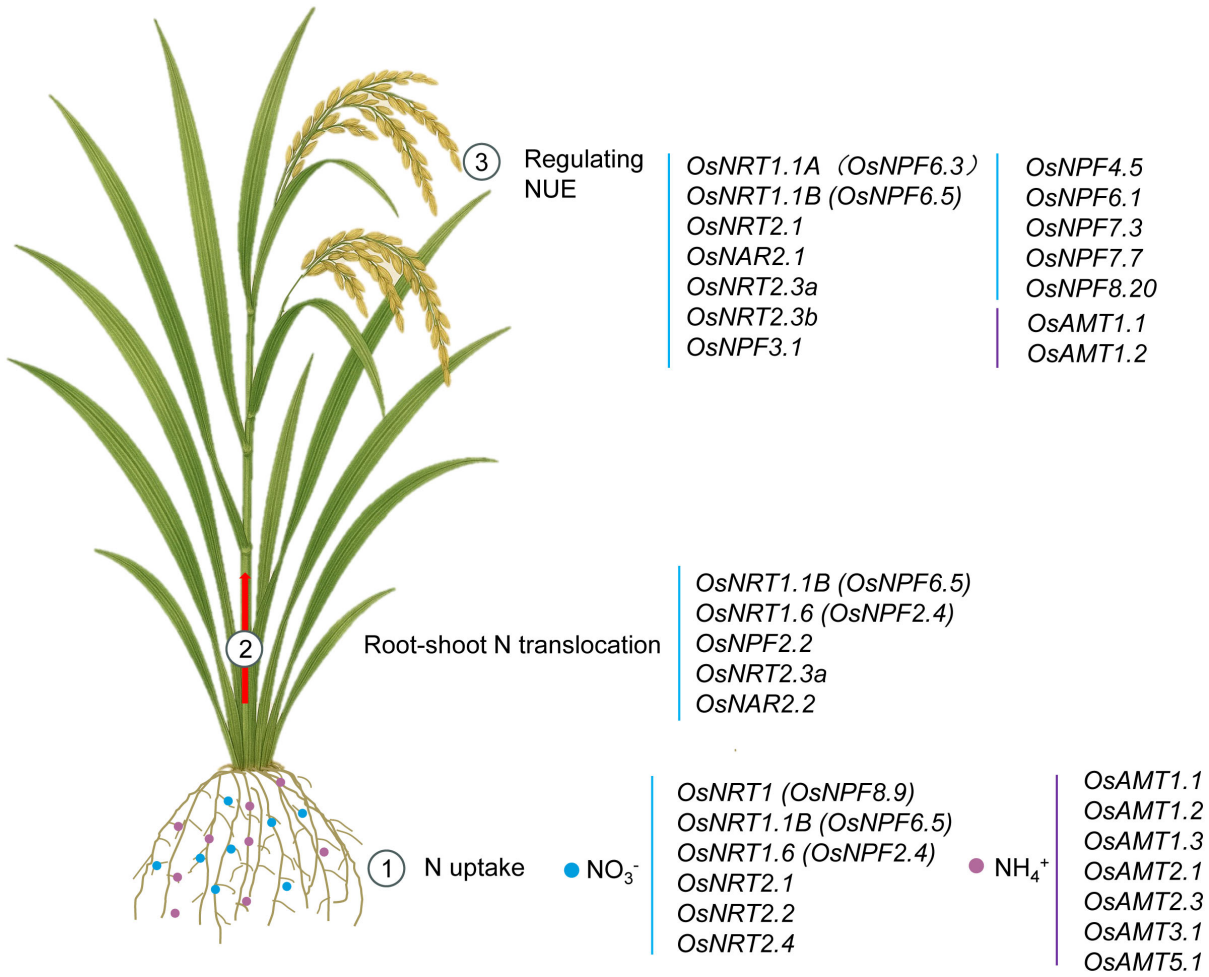
Integrative model to illustrate physiological functions of NO_3_^-^ and NH_4_^+^ transporters in rice. Detailed illustration of NO_3_^-^ and NH_4_^+^ uptake, translocation, and utilization in rice. Rice NO_3_^-^ uptake is orchestrated by transporters *OsNRT1*, *OsNRT1.1B*, *OsNRT1.6*, *OsNRT2.1*, *OsNRT2.2* and *OsNRT2.4*. For NH_4_^+^, *OsAMT1.1*, *OsAMT1.2*, *OsAMT1.3*, *OsAMT2.1*, *OsAMT2.3*, *OsAMT3.1* and *OsAMT5.1* are principal. *OsNRT1.1B*, *OsNRT1.6*, *OsNPF2.2*, *OsNRT2.3a* and *OsNAR2.2* are crucial for NO_3_^-^ translocation to shoots. Among the currently identified members of the rice NRT/NPF family, *OsNRT1.1A*, *OsNRT1.1B*, *OsNRT2.1*, *OsNAR2.1*, *OsNRT2.3a*, *OsNRT2.3b*, *OsNPF3.1*, *OsNPF4.5*, *OsNPF6.1*, *OsNPF7.7*, and *OsNPF8.20* have all been shown to enhance NUE, whereas *OsNPF7.3* decreased NUE at high NH_4_^+^ supply. In the AMT family, only *OsAMT1.1* and *OsAMT1.2* have been identified as capable of improving NUE.

Although NH_4_^+^ has long been recognized as the primary form of N absorbed by rice, research on NH_4_^+^ transporters have remained relatively limited. With the advancement of genomics, at least 12 potential NH_4_^+^ transporters (AMTs) have been identified in the rice genome. These transporters are classified into five subfamilies: *OsAMT1* (*OsAMT1.1*, *OsAMT1.2*, and *OsAMT1.3*), *OsAMT2* (*OsAMT2.1*, *OsAMT2.2*, and *OsAMT2.3*), *OsAMT3* (*OsAMT3.1*, *OsAMT3.2*, and *OsAMT3.3*), OsAMT4 (*OsAMT4.1*), and *OsAMT5* (*OsAMT5.1* and *OsAMT5.2*) (Suenaga et al., 2003; Li et al., 2009a). The *OsAMT1* subfamily functions as a high-affinity NH_4_^+^ transporter, operating under low NH_4_^+^ concentrations and exhibiting saturation kinetics. In contrast, the *OsAMT2*, *OsAMT3*, and *OsAMT4* families are classified as low-affinity transporters, predominantly active under high NH_4_^+^ concentrations (1–40 mM), and do not display saturation kinetics (Gaur et al., 2012). Studies have demonstrated that *OsAMT1.1*, *OsAMT1.2*, *OsAMT1.3*, *OsAMT2.1*, and *OsAMT5.1* all possess NH_4_^+^ transport capacity (Bu et al., 2011) (**Figure 1**).

### 2.2 Spatial and N-responsive expression of *OsAMTs*

The uptake of low concentrations of NH_4_^+^ in rice roots requires the coordinated activity of three *OsAMT1* members, which share high amino acid sequence homology. Among these, *OsAMT1.1* makes the largest contribution to N accumulation (Sonoda et al., 2003a; Zhao et al., 2014). Spatial expression analyses revealed that NH_4_^+^ exposure induces the upregulation of *OsAMT1.1* and *OsAMT1.2*, and the downregulation of *OsAMT1.3* (Konishi and Ma, 2021). *OsAMT1.1*, *OsAMT3.2*, and *OsAMT3.3* are constitutively expressed in both roots and stems, while *OsAMT1.1* expression is promoted by NH_4_^+^. *OsAMT1.2* is root-specific and induced by NH_4_^+^, whereas *OsAMT1.3* is root-specific but suppressed by N. *OsAMT2.1*, *OsAMT2.2*, *OsAMT2.3*, and *OsAMT3.1* are mainly expressed in aerial tissues, with relatively higher expression in stems than other genes. The expression of *OsAMT3* family genes is generally higher in shoots than in roots, suggesting that *AMT3* members may participate in the translocation and distribution of NH_4_^+^ within leaves (Suenaga et al., 2003; Gaur et al., 2012; Sonoda et al., 2003a; Li et al., 2012; Li and Shi, 2006). Additionally, the expression patterns of *OsAMT1* genes show strong correlations with Gln levels in root tissues (*OsAMT1.1* and *OsAMT1.2* positively correlated, *OsAMT1.3* negatively correlated), but not with NH_4_^+^ content (Sonoda et al., 2003b). *OsAMT5.1* is specifically expressed in leaves, with its expression enhanced by increasing NH_4_^+^ concentrations (Deng et al., 2007).

### 2.3 Functional divergence and regulatory mechanisms of key *OsAMT1* genes

At present, research on the regulation of AMT genes in rice has primarily focused on three *OsAMT1* family genes. Among them, *OsAMT1.1* plays a central role in N absorption and utilization in rice. It significantly promotes NH_4_^+^ uptake under both low and high NH_4_^+^ conditions, maintains N-potassium (K) homeostasis, and enhances NUE, plant growth, and grain yield under suboptimal to optimal N supply. Moreover, this gene underwent strong selection from wild rice to cultivated rice in response to soil conditions (**Figure 1**; **Table 1**) (Lee et al., 2020; Ranathunge et al., 2014; Ding et al., 2011). Simultaneous activation of *OsAMT1.2* and the glutamate synthase gene (*OsGOGAT1*) improve tolerance to N limitation and enhances NH_4_^+^ uptake and N remobilization at the whole-plant level (**Figure 1**; **Table 1**) (Lee et al., 2020). In contrast, overexpression of *OsAMT1.3* causes imbalances in carbon (C)-N metabolism, leading to poor plant growth and reduced yield (Bao et al., 2015).

**Table 1:** Overview of genes that are involved in regulation of NUE in rice.

Gene name	Effects to NUE and yield	Source	References
NO_3_^-^ transporters			
*OsNRT1.1A*	Improved NUE and promote flowering	Homologs of *Arabidopsis*	(Wang et al., 2018c)
*OsNRT1.1B*	Dual-affinity NO_3_^-^ transportation, modulated the root microbiome, influenced NUE in *indica* and *japonica*	Fine-mapping	(Hu et al., 2015; Zhang et al., 2019)
*OsNRT2.1*	Improved NUE and yield	Generate transgenic lines	(Chen et al., 2016)
*OsNAR2.1*	Enhanced NO_3_^-^ uptake, grain yield, and NUE	Generate transgenic lines	(Chen et al., 2017)
*OsNRT2.3a*	Co-overexpression of *OsNAR2.1* and *OsNRT2.3a* increased yield and NUE	Generate transgenic lines	(Chen et al., 2020)
*OsNRT2.3b*	Improved NUE and pH balance	Functional analysis	(Fan et al., 2016b)
*OsNPF3.1*	Increased NUE and biomass production	Fine-mapping	(Yang et al., 2023)
*OsNPF4.5*	Participated in mycorrhizal NO_3_^-^ acquisition	RNA Sequencing	(Wang et al., 2020a)
*OsNPF6.1*	*OsNPF6.1^HapB^* enhanced both NUE and yield	GWAS	(Tang et al., 2019)
*OsNPF7.3*	Increased plant growth at different N supplies but decreased NUE at high NH_4_^+^ supply	Generate transgenic lines	(Fan et al., 2014)
*OsNPF7.7*	Improved NUE and grain yield	Generate transgenic lines	(Huang et al., 2018)
*OsNPF8.20*	Improved NUE and grain yield	Homologs of *Arabidopsis*	(Fang et al., 2013)
NH_4_^+^ transporters			
*OsAMT1.1*	Improved NUE, plant growth, and grain yield	Phenotypic analysis	(Ranathunge et al., 2014)
*OsAMT1.2*	Concurrent activation of *OsAMT1.2* and *OsGOGAT1* enhanced NUE	Isolation of activation tagging mutants	(Lee et al., 2020)
Amino acid transporters or amino transferase			
*DNR1*	Involved in auxin homeostasis, reflects the differences in NO_3_^-^ uptake, N assimilation, and yield between *indica* and *japonica*	QTL	(Zhang et al., 2021b)
*ASL*	Coordinated regulation of NH_4_^+^ tolerance and NUE	MutMap and metabolomics analysis	(Xie et al., 2023)
*OsAAP1*	Regulation of spikelet fertility and NUE	Phenotypic analysis	(Pereira et al., 2022)
*OsAAP3*	Negative regulation of NUE and yield	Haplotype analysis	(Lu et al., 2018)
Transcription factor			
*SOD5*	Knocking out *SOD5* significantly increases NUE and grain yield	Identify upstream regulators	(Zhang et al., 2025a)
*OsWRKY23*	A key regulator of NO_3_^-^ uptake and NUE differences between *indica* and *japonica*	Fine-mapping	(Zhang et al., 2025b)
*OsMYB61*	Promotes N utilization and biomass production	QTL and map-based cloning	(Gao et al., 2020)
*OsNAC42*	Improved NUE	GWAS	(Tang et al., 2019)
*OsNLP4*	Improved NUE and yield	GWAS, generate transgenic lines	(Yu et al., 2021; Wu et al., 2021)
*OsTCP19*	*OsTCP19^H^* holds the potential for improving NUE	GWAS	(Liu et al., 2021)
*OsGATA8*	Natural variation in the *OsGATA8* promoter influences NUE	GWAS	(Wu et al., 2024)
*ARE4*	MYB-related transcription factor, coordinated regulation of glucose signaling and NUE	Ethyl methane sulfonate mutagenesis	(Ma et al., 2023)
*OsDOF18*	Mediated NH_4_^+^ transport and N distribution, affected NUE	T-DNA insertion mutant	(Wu et al., 2017)
*OsDREB1C*	Improved crop yields and NUE, and promoted earlier flowering	Transcriptomes and metabolomes	(Wei et al., 2022)
*OsRF2b*	Negative regulation of NUE and yield	Biochemical screening methods	(Li et al., 2025)
*OsbZIP61*	Negative regulation of NUE and yield	N-relative gene expression variations	(Li et al., 2025)
*GRF4*	Improved NUE and grain yield in Green Revolution varieties	QTL	(Li et al., 2018)
*NGR5*	Improved NUE and grain yield	Ethyl methane sulfonate mutagenesis, map-based cloning	(Wu et al., 2020)
Enzymes for N assimilation and remobilization			
*OsNR2*	Increased effective tiller number, grain yield and NUE	QTL	(Gao et al., 2019)
*OsNR1.2*	Encode an NADH-dependent NO_3_^-^ reductase that is required for high NUE	Reverse-transcription quantitative PCR	(Han et al., 2022)
Others			
*OsSTP28*	Encode an influx hexose transporter, modulated N-determined tillering and yield formation	GWAS	(Zhang et al., 2024)
*ARE1*	*abc1–*1 repressor, mediated grain yield by modulating NUE	Mutant genetic screen	(Wang et al., 2018b)
*OSA1*	Plasma membrane H^+^-ATPase, cooperatively improve N and C utilisation	Phenotypic analysis	(Zhang et al., 2021a)
*OsBT1/BT2*	Increased NUE by 20% compared to wild-type	Homologs of *Arabidopsis*	(Araus et al., 2016)
*RNR10*	Causal genes with that underlies the significantly different root developmental plasticity in response to changes in N level exhibited by the *indica* and *japonica*	Fine-mapping	(Huang et al., 2023)

### 2.4 Perspectives on the regulatory and metabolic roles of *AMT* genes in rice

The AMT gene family plays a critical role in NH_4_^+^ uptake in rice. To date, studies on the phylogeny, expression patterns, and functions of rice AMT genes have provided preliminary insights into their roles in N absorption. However, the regulation of AMT genes is not limited to N uptake but may also be involved in N assimilation, translocation, and other N-related metabolic processes. Therefore, future studies should expand our understanding of AMT gene regulation, environmental adaptability, and especially their integrative roles in N transport and metabolism. Such research will help uncover the potential applications of these genes in improving NUE and crop productivity in rice, ultimately offering new strategies for sustainable agricultural production and environmental protection.

## 3 Functions of NO_3_^-^ transporters in rice

The absorption of NO_3_^-^ is an active process driven by H^+^/NO_3_^-^ co-transporters (Miller et al., 2007). Although rice is a plant that prefers NH_4_^+^, under the action of soil microorganisms, NH_4_^+^ can be converted into NO_3_^-^ through nitrification. In addition, N fertilizers applied to the soil are also partially converted into NO_3_^-^, which can then be absorbed and utilized by rice. As a result, about 25-40% of the total N absorbed by rice exists in the form of NO_3_^-^ (Xu et al., 2012; Li et al., 2008; Kirk and Kronzucker, 2005). Compared with NH_4_^+^, the mechanisms of NO_3_^-^ absorption have been more extensively studied in rice, and the corresponding transporters have been thoroughly identified. In rice, NO_3_^-^ transporters are generally classified into two families: the low-affinity *NRT1* family and the high-affinity *NRT2* family, enabling rice to adapt to changes in N availability in the environment.

### 3.1 NRT1/PTR family: low-affinity NO_3_^-^ transporters

The number of NO_3_^-^ transporter 1/peptide transporter (NRT1/PTR, also known as NPF) family members in rice has been confirmed by several genomic analyses, with approximately 93 NPF genes identified. However, the functions of only a few NPF family members have been characterized to date (Léran et al., 2014). Different members of the NPF family perform distinct functions in rice. *NRT1.1* in rice, an important NO_3_^-^ transporter belonging to the *NPF6* subfamily, mainly includes three homologs: *OsNRT1.1A* (*OsNPF6.3*), *OsNRT1.1B* (*OsNPF6.5*), and *OsNRT1.1C* (*OsNPF6.4*), which play critical roles in N uptake, transport, signaling, and NUE (Wang et al., 2020b).

**Figure 2:**
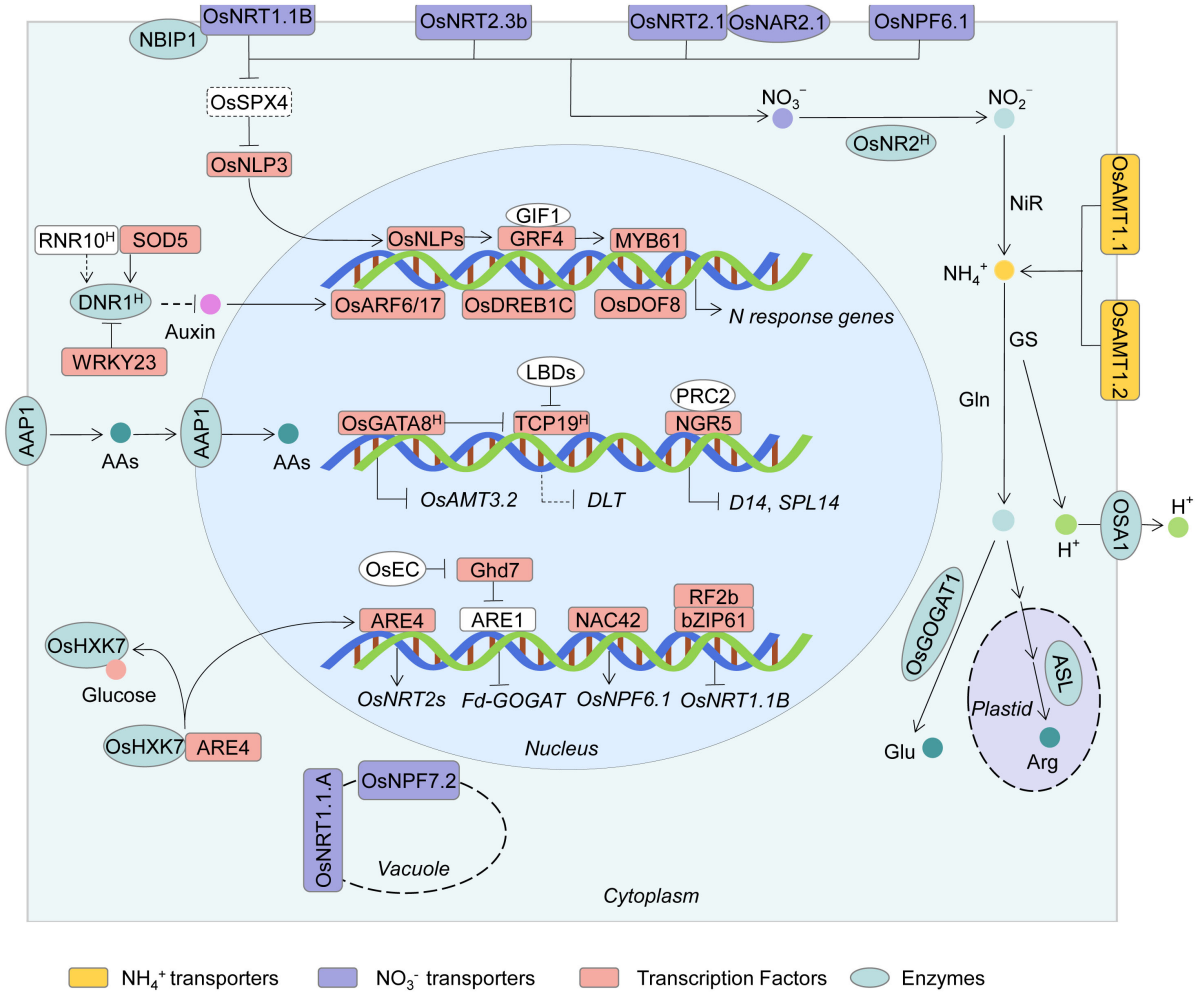
Schematic representation of key regulatory pathways and major genes involved in NUE in rice. This model summarizes major molecular players and signaling pathways involved in NO_3_^-^ and NH_4_^+^ uptake, assimilation, and systemic regulation of NUE in rice. NO_3_⁻ and NH_4_^+^ are absorbed via NRT/NPF and AMT transporter families, respectively. A central NO_3_^-^ signaling module comprising OsNRT1.1B-OsSPX4-OsNLP3/NLP4 integrates NO_3_^-^ sensing and transcriptional responses, regulating genes involved in N uptake and assimilation. Key transcription factors, including OsDREB1C, GRF4, NGR5, WRKY23, GATA8, and ARE4, modulate NUE through direct or indirect regulation of transporter genes and metabolic enzymes. Auxin-mediated pathways also contribute to NUE control: DNR1 antagonizes auxin accumulation and NO_3_⁻ responsiveness, while RNR10, SOD5, and WRKY23 regulate DNR1 expression or stability. The GRF4-MYB61, Ghd7-ARE1, and NGR5-PRC2 modules further coordinate NUE with plant development and chromatin dynamics. Additional regulators include OsDOF18, which activates *AMT* genes, OsGATA8 and OsTCP19, which link N status to tillering, and bZIP transcription factors OsRF2b/OsbZIP61, which negatively regulate *OsNRT1.1B*. The amino acid transporter OsAAP1, located on both the plasma membrane and nuclear membrane, is capable of absorbing and transporting amino acids, thereby enhancing NUE. Together, these components form a complex regulatory network integrating nutrient signaling, transcriptional control, hormone crosstalk, and epigenetic regulation to optimize NUE and sustain rice yield under variable N inputs.

*OsNRT1.1A* exhibits NH_4_^+^-induced expression and can significantly upregulate the expression of various genes related to NO_3_^-^ and NH_4_^+^ utilization. Overexpression of *OsNRT1.1A* significantly improves NUE and grain yield and also shortens the rice maturity period, providing a feasible approach for breeding high-yield, early-maturing rice varieties (Wang et al., 2018c). Among the *NRT1* family members in rice, only *OsNRT1.1B* possesses dual-affinity transport properties and functions across a wide range of NO_3_^-^ concentrations. Under low N (LN) conditions, *OsNRT1.1B* enables plants to accumulate more N and promotes rice growth, whereas *OsNRT1.1A* lacks such functionality in rice (Fan et al., 2016a). The NO_3_^-^ uptake activity of *indica* rice is higher than that of *japonica*, and genetic variation in *OsNRT1.1B* significantly influences differences in NUE between *indica* and *japonica* by regulating NO_3_^-^ uptake and rhizosphere microbiota. Moreover, introducing the *NRT1.1B^indica^* allele into *japonica* could potentially enhance the NUE of *japonica* rice (Hu et al., 2015; Zhang et al., 2019) (**Figure 1**; **Figure 2**; **Table 1**). The functional differentiation between OsNRT1.1A and OsNRT1.1B helps rice coordinate internal and external N signals and improve its adaptability to complex N environments (Wang et al., 2020b). *OsNRT1.3* promoter responds to drought stress, potentially participating in basic NO_3_^-^ uptake and stress responses (Hu et al., 2006).

Within the rice NPF family, *OsNPF2.2* can unload NO_3_^-^ from the xylem, thereby affecting NO_3_^-^ transport in the root-stem and plant development (Li et al., 2015). *OsNPF2.4* (*OsNRT1.6*) is a pH-dependent low-affinity transporter functioning in NO_3_^-^ uptake, long-distance transport, and redistribution, while its altered expression indirectly affects K reutilization in roots and stems (Xia et al., 2015). A coding region mutation in *OsNPF3.1* affects NUE differences between wild and cultivated rice and can improve NUE and biomass yield (Yang et al., 2023). *OsNPF4.1* (*SP1*) encodes a putative peptide transporter highly expressed in the phloem of young panicle branches, controlling panicle size (Li et al., 2009b). Mycorrhizal rice could receive more than 40% of its N via the mycorrhizal pathway, and the arbuscular mycorrhizal-specific NO_3_^-^ transporter *OsNPF4.5* accounted for approximately 45% of the mycorrhizal NO_3_^-^ uptake. Enhanced expression of *NPF4.5* can significantly improve NUE and promote rice growth (Wang et al., 2020a). The NO_3_^-^ transporter *OsNPF5.16* positively regulates rice tillering and yield by regulating cytokinin levels (Wang et al., 2022). *OsNPF6.1* is NO_3_^–^inducible and has two haplotypes: *OsNPF6.1^HapA^* and *OsNPF6.1^HapB^*. *OsNPF6.1^HapB^* enhances NO_3_^-^ absorption and improves NUE. Furthermore, *OsNPF6.1* enhances viral resistance by upregulating the expression of NO_3_^-^ reductase (OsNR2) and subsequently promoting nitric oxide (NO) biosynthesis (Tang et al., 2019; Xu et al., 2025) (**Figure 1**; **Table 1**).

Among the *OsNPF7* and *OsNPF8* subfamilies, *OsNPF7.1* (*OsPTR4*) and *OsNPF7.4* show opposite expression patterns in tiller buds under different N concentrations. Overexpression of either *OsNPF7.1* or *OsNPF7.4* promotes NO_3_^-^ absorption, although biomass is reduced in *OsNPF7.4*-overexpressing plants (Huang et al., 2019b). *OsNPF7.2* acts as a positive regulator of NO_3_^-^ influx and concentration, with overexpression lines showing significant increases in tiller number and yield (Hu et al., 2016; Wang et al., 2018a). *OsNPF7.3* (*OsPTR6*) mainly transports di- and tripeptides (e.g., Gly-His, Gly-His-Gly). While its overexpression promotes rice growth, its effect on NUE is limited (Fan et al., 2014); however, subsequent studies revealed that overexpression of both *OsNPF7.3* and *OsNPF7.7* increases rice tiller number, NUE, and yield (Fang et al., 2017; Huang et al., 2018). *OsNPF8.1* (*OsPTR7*) mediates stress-induced organic N transport, contributing to balanced plant growth and enhanced tolerance to salt/drought stress and N deficiency (Qiu et al., 2023). *OsNPF8.9* (*OsNRT1*) is the first low-affinity NO_3_^-^ transporter identified in rice, functioning under high NO_3_^-^ conditions (Lin et al., 2000). The di-/tripeptide and low-affinity NO_3_^-^ transporter *OsNPF8.20* (*OsPTR9*) enhances NH_4_^+^ uptake, promotes lateral root formation, and increases grain yield when its expression is upregulated (Fang et al., 2013) (**Figure 1**; **Table 1**).

### 3.2 NRT2/NAR2 family: high-affinity NO_3_^-^ transporters

The HATS play a crucial role in rice N uptake. To date, five *NRT2* (*OsNRT2.1*, *OsNRT2.2*, *OsNRT2.3a*, *OsNRT2.3b*, *OsNRT2.4*) and two *NAR2* (*OsNAR2.1*, *OsNAR2.2*) high-affinity NO_3_^-^ transporters have been identified in rice (Araki and Hasegawa, 2006; Feng et al., 2011; Cai et al., 2008). The rice *NRT2* and *NAR2* family members exhibit distinct functions. Some *NRT2* members require the partner protein *NAR2* for NO_3_^-^ transport within relatively low concentration ranges (Cai et al., 2008).

*OsNRT2.3* generates two transcripts, *OsNRT2.3a* and *OsNRT2.3b*, through mRNA splicing, with 94.2% amino acid sequence identity and identical coding regions but different 5′ and 3′ untranslated regions. *OsNRT2.3a* is primarily expressed in roots and induced by NO_3_^-^, whereas *OsNRT2.3b* is mainly expressed in shoots. Further research revealed that under LN supply, *OsNRT2.3a* plays a key role in long-distance NO_3_^-^ transport from roots to shoots, and *OsMADS57* regulates NO_3_^-^ transport through *OsNRT2.3a* (Huang et al., 2019a; Tang et al., 2012). Allelic variation in the 5’ untranslated region of *OsNRT2.3* leads to elevated *OsNRT2.3b* protein levels under high-temperature stress, increasing yield (Zhang et al., 2022). Additionally, high *OsNRT2.3b* expression enhances pH buffering capacity and improves the uptake of N, iron, and phosphorus (Fan et al., 2016b; Feng et al., 2017).

OsNAR2.1 interacts with OsNRT2.1/2.2 and OsNRT2.3a to mediate NO_3_^-^ uptake (Feng et al., 2011; Yan et al., 2011). OsNAR2.2, localized to the endoplasmic reticulum (ER), was recently shown to regulate NO_3_^-^ transport from roots to stems and control spikelet number, yield, and NUE in rice (Hou et al., 2025). *OsNRT2.1*, *OsNRT2.2*, and *OsNAR2.1* are promising candidate genes for breeding high NUE rice cultivars (Araki and Hasegawa, 2006). Enhancing *OsNAR2.1* expression via its native promoter, or increasing *OsNRT2.1* expression under the control of the *OsNAR2.1* promoter, or co-overexpressing *OsNAR2.1* and *OsNRT2.3a* can all improve NO_3_^-^ uptake, yield, and NUE in rice (**Figure 1**; **Table 1**) (Chen et al., 2016, Chen et al., 2017, Chen et al., 2020). *OsNRT2.4*, a dual-affinity NO_3_^-^ transporter, participates in regulating NO_3_^-^ uptake and allocation between roots and shoots and promotes plant growth and development under NO_3_^-^ regulation (Wei et al., 2018).

### 3.3 NO_3_^-^ sensing and signal transduction

In rice, NO_3_^-^ acts not only as a nutrient but also as a signaling molecule. OsNRT1.1B has been confirmed to sense external NO_3_^-^ signal (Hu et al., 2015). Additionally, studies have shown that the NO_3_^-^ sensor OsNRT1.1B physically interacts with the phosphate signaling repressor OsSPX4; the presence of NO_3_^-^ enhances this interaction and promotes the recruitment of *NRT1.1B*-Interacting Protein 1 (*NBIP1*, an E3 ubiquitin ligase), leading to the ubiquitination and degradation of OsSPX4. The core NO_3_^-^ signaling transcription factor *NLP3* is also regulated by SPX4. This OsNRT1.1B-OsSPX4-OsNLP3 regulatory module fills the gap between plasma membrane NO_3_^-^ sensing and downstream NO_3_^-^ responses in the nucleus (**Figure 2**) (Wang et al., 2020b; Hu et al., 2019).

## 4 Key genes regulating nitrogen use efficiency in rice

### 4.1 NUE-associated genes identified by QTL and map-based cloning

During crop domestication, many advantageous variant loci are retained by natural or artificial selection. Identifying these natural variant loci can provide theoretical support for crop genetic improvement (Hu et al., 2015; Li et al., 2018). In modern rice cultivars, NUE-related quantitative trait loci (QTL) or genes have been identified through map-based cloning methods. According to the varying N absorption capacity among different varieties, key genes controlling NUE, such as *OsNRT1.1B*, *OsNR2*, *DNR1*, and *OsWRKY23*, have been cloned (Hu et al., 2015; Li et al., 2018; Zhang et al., 2021b; Gao et al., 2019; Zhang et al., 2025b).

*OsNRT1.1B* and *OsNR2* in *indica* have significant improvement in NUE and grain yield than those in *japonica* (Hu et al., 2015; Gao et al., 2019). Auxin Response Factor OsARFs mediate the promotion of N metabolism by auxin, DNR1 participates in the regulation of auxin homeostasis, and reflects differences in NO_3_^-^ uptake, N assimilation, and yield enhancement between *indica* and *japonica*. The variation in the promoter of *DNR1* in *indica* decreased expression levels and a higher auxin content, which triggers ARF-activated the transcription of NO_3_^-^ uptake and assimilation-related genes, leading to improving the grain yield and NUE (Zhang et al., 2021b). RNR10 encodes an F-box protein that interacts with DNR1. RNR10 monoubiquitinates DNR1 and inhibits its degradation, thus antagonizing auxin accumulation, which results in reduced root responsivity to N and NO_3_^-^ uptake (Huang et al., 2023). SOD5 directly binds to the *DNR1* promoter, activates its expression, and further inhibits auxin accumulation. Notably, knockout of *SOD5* significantly improves NUE and grain yield, especially under LN conditions (Zhang et al., 2025a). OsWRKY23 is a key regulator of the differences in NO_3_^-^ absorption rate and NUE between *indica* and *japonica* rice. *OsWRKY23^indica^* exhibits reduced transcriptional activation of *DNR1*, leading to higher auxin levels, improved NO_3_^-^ absorption and assimilation, and ultimately enhanced NUE and yield (Zhang et al., 2025b).

Different varieties exhibit developmental differences due to their varying sensitivity to N supply, which can be observed in factors such as root length, biomass, and yield. Map-based cloning has been used to identify the genetic loci responsible for N modulation of plant growth, such as *MYB61*, through analysis of phenotypic values of these traits or ratios under different N levels supplied (Gao et al., 2020; Obara et al., 2010; Lian et al., 2005). The transcription factor *MYB61* is regulated by GROWTH-REGULATING FACTOR4 (GRF4) and coordinates the production of cellulosic biomass and N utilization. The *indica* allele of *MYB61* shows strong transcriptional activity, leading to improved NUE and higher grain yield under reduced N supply compared to the *japonica* allele (Gao et al., 2020) (**Figure 2**; **Table 1**).

### 4.2 NUE-associated genes identified by GWAS

In recent years, genome-wide association study (GWAS) has also been used to locate some N-efficient genes. For example, using NUE-related agronomic traits, the GWAS identified the excellent variation of *OsNPF6.1^HapB^*, which originated from the variation of wild rice. This excellent allele was transcriptionally activated by NAC42, and it enhances the ability of N absorption capacity and improves NUE under LN conditions (Tang et al., 2019). Through GWAS analysis of NUE-related traits (effective panicle number and yield per plant) in natural populations of rice, combined with transcript data under high N (HN) and LN conditions, OsNLP4 was identified as a transcription factor that regulates NUE. Simultaneously, OsNLP4 can promote the transcription of nitrite reductase gene *OsNiR* and N transport-related genes (*NRTs and AMT1.1*) (Yu et al., 2021; Wu et al., 2021), achieving coordinated regulation of N uptake and assimilation in rice. Accordingly, the localization of OsNLP3 and OsNLP4 in rice cells is affected by NO_3_^-^ supply levels. With the application of NO_3_^-^, the localization of OsNLP3 and OsNLP4 in cells is shifted from cytoplasm to nucleus (Hu et al., 2019; Wu et al., 2021), indicating that OsNLP3 and OsNLP4 are central regulatory factors in the N signaling pathway of rice.

Using a multiparent advanced generation intercross (MAGIC) population, GWAS of NUE-related traits (tillering) under HN and LN conditions identified the OsSTP28 as a key regulator of N-responsive tillering and yield formation in rice (Zhang et al., 2024). A GWAS for N-responsive tillering in rice identified OsTCP19 as a regulatory factor; a 29-bp indel in the promoter of *OsTCP19* represents a natural variation that determines tiller number under LN conditions in different rice varieties. OsTCP19 as a modulator of tillering response to N through its transcriptional response to N and its targeting to the tiller-promoting gene *DWARF AND LOW-TILLERING* (*DLT*), the OsLBD37/39-OsTCP19-DLT pathway is a key regulatory cascade governing N response and tillering in rice (Liu et al., 2021). Furthermore, the transcription factor OsGATA8 was identified as a critical regulator of N uptake and tiller formation in rice. OsGATA8 negatively regulates N absorption by repressing *OsAMT3.2* transcription, while promoting tiller formation by inhibiting the transcription of the negative tillering regulator *OsTCP19*. The *OsGATA8^H^* haplotype displays high NUE, with enhanced N uptake and a higher proportion of productive tillers (Wu et al., 2024) (**Figure 2**; **Table 1**).

### 4.3 NUE-associated genes identified by mutant identification

In recent years, several NUE-related genes have been cloned from mutant identification, such as *ARE1*, *ARE4*, *OsELF3-1*, and *OsDOF18*. *ARE1* is a negative regulator of N assimilation, encoding a chloroplast-localized protein, and is transcriptionally inhibited by Ghd7. Loss-of-function mutations in *ARE1* cause delayed senescence and grain yield increases, hence enhance NUE under LN conditions (Wang et al., 2018b). OsELF3–1 forms a ternary complex (OsEC) with OsELF4s and OsLUX, repressing the expression of *Ghd7*, which in turn directly inhibits *ARE1* expression and promotes N absorption (Wang et al., 2021; Tsednee, 2024; Sun et al., 2024). ARE4, a MYB-related transcription factor, coordinates glucose signaling with NUE in rice. It is kept in the cytosol by interacting with the glucose sensor OsHXK7. Upon sensing a glucose signal, ARE4 is released, translocated into the nucleus, and activates the expression of a group of high-affinity NO_3_^-^ transporter genes, resulting in increased NO_3_^-^ uptake and accumulation (Ma et al., 2023). In the *osdof18* mutant, the expression of *OsAMT1.1*, *OsAMT1.3*, *OsAMT2.1*, and *OsAMT4.1* is reduced, indicating that these NH_4_^+^ transporter genes function downstream of the transcription factor OsDOF18. The findings demonstrate that OsDOF18 mediates NH_4_^+^ transport and N allocation, thereby influencing NUE (Wu et al., 2017) (**Figure 2**; **Table 1**).

### 4.4 Regulation of cellular pH homeostasis enhances NUE in rice

Excessive absorption of NH_4_^+^ by plants leads to cellular acidification, while excessive NO_3_^-^ uptake leads to cellular alkalization. Therefore, excessive uptake of a single N source affects the pH balance in plant cells, causing enzyme dysfunction and ultimately impacting crop growth and yield. In a recent study, overexpression of the N transport gene *OsNRT2.3b* helps counteract pH changes in rice plants, thus improving NUE and rice yield (Fan et al., 2016b). Plasma membrane (PM) H^+^-ATPase facilitates the transport of various nutrients, such as NO_3_^-^, phosphate (Pi), and K, and maintains cytosolic H^+^ homeostasis by pumping H^+^ outside the cells. In previous studies, overexpression of *Oryza sativa* PM H^+^-ATPase 1 (*OSA1*) in rice enhances NH_4_^+^ uptake and assimilation, leading to increased grain yield and NUE (Zhang et al., 2021a). The assimilation of NH_4_^+^ in root cells requires a C skeleton as the substrate for the synthesis of amino acids through the GS/GOGAT cycle. The assimilation of one molecule of NH_4_^+^ generates two molecules of H^+^ in the cytoplasm. This inhibits the growth and development of plant roots and reduces NUE (Jia et al., 2020; Hachiya et al., 2021). A recent study identified a mutant that exhibited root hypersensitivity to NH_4_^+^ due to a missense mutation in the gene encoding argininosuccinate lyase (ASL), which localizes to plastids and mitigates NH_4_^+^-induced inhibition of root elongation by converting excess glutamine into arginine. Natural variations in *ASL* alleles between the *japonica* and *indica* subspecies of rice demonstrate *ASL* expression is positively correlated with NUE and yield (Xie et al., 2023). These results suggest that the H^+^ produced during the mitigation of NH_4_^+^ assimilation can improve NUE in rice (**Figure 2**; **Table 1**).

### 4.5 Other key regulatory genes involved in NUE in rice

The transcription factor OsDREB1C is identified through RNA-seq as co-induced by light and LN supply, directly targets *OsNR2*, *OsNRT2.4*, and *OsNRT1.1B*, and simultaneously enhances the efficiency of photosynthesis and NUE, significantly improving rice yield (Wei et al., 2022). The bZIP transcription factor OsRF2b, identified through biochemical screening, interacts with OsbZIP61 to form heterodimers. This complex directly binds to the *OsNRT1.1B* promoter region and represses its expression, acting as a negative regulator of NUE and grain yield (Li et al., 2025). OsNR1.2 encodes an NADH-dependent NO_3_^-^ reductase, essential for achieving high NUE in rice (Han et al., 2022). Furthermore, a batch of key genes involved in NUE has also been identified, such as rice *OsBT1*, *OsBT2*, and *AAP* genes (Araus et al., 2016; Lu et al., 2018; Pereira et al., 2022), crop yield and NUE can be improved by changing the expression levels of these genes (**Figure 2**; **Table 1**).

### 4.6 Dissecting NUE pathways in green revolution varieties

Apart from using the methods mentioned above to identify N-efficient genes, analyzing the mechanism of low NUE in ‘Green Revolution’ varieties (GRVs) that limit efficient N use, and mining N-efficient genes from such varieties have also proven effective. The ‘Green Revolution’ gene *sd1*, which encodes the GA20 oxidase 2 (GA20ox2) enzyme, an important synthetic enzyme in the gibberellin (GA) synthesis pathway, is widely used in indica breeding. The mutated type of *sd1* causes a decrease in endogenous GA activity and GA signal suppressor DELLAs protein (SLR1) accumulation in rice, which leads to a reduction in rice plant height (Ashikari et al., 2002).

A rice transcription factor GRF4 interacts with the transcriptional activator GIF1 to promote the expression of N transport and assimilation-related genes (such as *OsAMT1.1*, *OsGS1.2*, *OsNRT1.1B*) (Li et al., 2018). SLR1 competitively represses the GRF4-GIF1 interaction, inhibiting the formation of the GRF4-GIF1 protein complex, which leads to a reduction in NUE of rice. Introduction of the excellent allele gene *GRF4^ngr2^* into semi-dwarf and high-yielding rice varieties can achieve a coordinated increase in the yield and NUE of rice without changing their plant height (Li et al., 2018). The key repressor DWARF 53 (D53) of the SL signalling interacts with GRF4 and prevents GRF4 from binding to its target gene promoters, and negatively regulates NUE (Sun et al., 2023).

Subsequently, NGR5 is a new target of GA-GIBBERELLIN-INSENSITIVE DWARF1 (GID1)-mediated proteasomal destruction, and SLR1 competes with NGR5 for interaction with GID1, in the case of NGR5, with stabilized SLR1 of rice GRVs promoting stabilization of NGR5, thus explaining why GRVs exhibit increased tillering. N status affects chromatin function through modification of histones, a process in which the transcription factor NGR5 recruits polycomb repressive complex 2 (PRC2) to inhibit tiller genes, including *OsD14* and *OsSPL14*, through repressive H3K27me3 modifications (Wu et al., 2020). Additionally, SLR1 competes with NGR5 for interaction with GID1. In the case of NGR5 with stabilized SLR1 of rice (GRVs), promoting the stabilization of NGR5 leads to increased tillering, explaining why GRVs exhibit enhanced tillering. Furthermore, pyramiding of *sd1* elite NGR5 alleles can enhance NUE, leading to reduced N fertilizer usage and increased grain yield, without affecting the beneficial semi-dwarfism (Wu et al., 2020) (**Figure 2**; **Table 1**). This suggests that manipulation of plant development and NUE co-modulation would drive modern breeding for sustainable food security.

## 5 Conclusion and future perspectives

### 5.1 Identification of key NO_3_^-^ and NH_4_^+^ transporters enhancing NUE in rice

Over the past two decades, one of the most significant advances in understanding N utilization regulation in rice has been the identification of NO_3_^-^ and NH_4_^+^ transporters, as well as transcription factors involved in NUE. In rice, *OsNRT1*, *OsNRT1.1B*, *OsNRT1.6*, *OsNRT2.1*, *OsNRT2.2* and *OsNRT2.4* are responsible for NO_3_^-^ uptake. *OsAMT1.1*, *OsAMT1.2*, *OsAMT1.3*, *OsAMT2.1*, *OsAMT2.3*, *OsAMT3.1* and *OsAMT5.1* are principal for NH_4_^+^ uptake. *OsNRT1.1B*, *OsNRT1.6*, *OsNPF2.2*, *OsNRT2.3a* and *OsNAR2.2* are crucial for NO_3_^-^ translocation to shoots. In addition, among the currently identified members of the rice NRT/NPF family, *OsNRT1.1A*, *OsNRT1.1B*, *OsNRT2.1*, *OsNAR2.1*, *OsNRT2.3a*, *OsNRT2.3b*, *OsNPF3.1*, *OsNPF4.5*, *OsNPF6.1*, *OsNPF7.7*, and *OsNPF8.20* have all been shown to enhance NUE, whereas *OsNPF7.3* decreased NUE at high NH_4_^+^ supply. Compared to the substantial advances made in NO_3_^-^ transporters, the progress achieved with NH_4_^+^ transporter proteins in improving NUE has remained relatively limited. In the AMT family, only *OsAMT1.1* and *OsAMT1.2* have been identified as capable of improving NUE (**Figure 1**; **Table 1**).

### 5.2 Genetic and molecular strategies for enhancing NUE in rice

Significant genetic variation in NUE exists within rice germplasm resources, providing a valuable foundation for the precise breeding of cultivars with enhanced NUE. During rice domestication, natural variations in several key loci genes have been identified (including *OsNRT1.1B*, *OsNR2*, *DNR1*, *OsWRKY23*, and *MYB61*), playing important roles in regulating NUE and contributing to yield differences between indica and japonica cultivars. In the future, it remains essential to further dissect the candidate genes responsible for NUE variation among rice germplasm resources. GWAS has already cloned multiple key NUE-regulating genes, such as *OsNPF6.1*, *OsNLP4*, *OsSTP28*, *OsTCP19*, and *OsGATA8*, which enhance N uptake, assimilation, and tillering ability in rice, thereby improving both NUE and grain yield under varying N conditions. These findings have provided important genetic resources for the molecular breeding of rice cultivars with improved NUE. Moreover, maintaining cellular pH homeostasis is crucial for achieving high NUE in rice. Genes such as *OsNRT2.3b*, *OSA1*, and *ASL* play pivotal roles in regulating proton flux, nutrient transport, and N assimilation, ultimately enhancing NUE and grain yield under N stress conditions. Moreover, introducing advantageous alleles such as *GRF4^ngr2^* or *NGR5* into the genetic background of the ‘Green Revolution’ *sd1* can simultaneously enhance NUE and grain yield while maintaining a desirable dwarf plant architecture. The synergistic effect of sd1-GRF4-NGR5 enables the coordinated improvement of both NUE and yield, representing an ideal strategy for future molecular breeding (**Figure 2**; **Table 1**).

### 5.3 Multi-gene co-regulation strategies for enhancing NUE in rice

Despite the successive identification and characterization of key genes involved in N uptake, transport, and utilization in rice, the effect of a single gene on improving N absorption or NUE remains limited. By adopting a multi−gene co−regulation strategy, rice NUE and yield can be further enhanced. Studies have shown that co−overexpression of genes for N transport, uptake, and assimilation increases both rice yield and NUE. For instance, the *OsNPF8.9a*×*OsNR2*, *OsAMT1.2*×*OsGS1.2*×*OsAS1*, and *OsGS2*×*OsAS2*×*OsANT3* combinations respectively optimize NO_3_^-^ uptake, NH_4_^+^ conversion, and N recycling. Notably, combining *OsAMT1.2*, *OsGS1.2*, and *OsAS1* overexpression represents a promising breeding strategy (Luo et al., 2023).

Looking forward, the success of such multi-gene strategies will benefit greatly from the integration of advanced biotechnological tools. CRISPR/Cas-based multiplex genome editing allows for precise and simultaneous modification of multiple target genes, while transgenic stacking enables coordinated expression of gene cassettes. When combined with high-throughput phenotyping and omics-assisted selection, these approaches provide a robust framework for the rational design of rice cultivars with enhanced NUE and yield potential. This systems-level breeding strategy offers an effective path toward reducing N fertilizer input while maintaining high productivity, contributing to more sustainable and environmentally friendly rice production.

### 5.4 Integration of NUE regulation with environmental stress responses

In the context of global climate change and increasingly variable field conditions, the regulation of NUE in rice must be understood not only under optimal environments but also under abiotic stress conditions. Recent studies have demonstrated that N uptake and assimilation are not only genetically regulated but also highly responsive to environmental cues. Under abiotic stresses such as drought, salinity, and extreme temperatures, N transporter expression and NR activity or other enzymes involved in N metabolism are frequently suppressed, leading to reduced NUE (Henckel, 1964; Plaut, 1974; Guo et al., 2003; Robredo et al., 2011; Goel and Singh, 2015; Han et al., 2015; Meng et al., 2016).

However, the application of key genes has been shown to be significant potential for improving NUE under stress conditions. For instance, DST (Drought and Salt Tolerance)-OsNR1.2 regulatory module has been shown to be involved in the suppression of NO_3_^-^ assimilation under drought tolerance. Given that DST negatively regulates stomatal closure while positively regulating N assimilation, it likely mediates a coupling between N metabolism and stomatal movement. This mechanism offers a promising target for developing drought-tolerant crops with improved NUE (Han et al., 2022). OsDREB1C, a member of the AP2/EREBP transcription factor family, was initially identified for its role in cold stress responses in rice, recent studies have demonstrated that overexpression of *OsDREB1C* shortens the growth duration, enhances NUE, and promotes more effective resource allocation, suggesting a potential regulatory link between stress response pathways and nutrient efficiency (Mao and Chen, 2012; Wei et al., 2022).

Moreover, agronomic practices such as irrigation regimes, fertilization strategies, and soil amendments can influence the expression and function of key N-related genes, thereby affecting NUE in crops (Shoji et al., 2001; Yang et al., 2015; Sajjad et al., 2024). Furthermore, in paddy fields, NO_3_^-^ and NH_4_^+^ availability fluctuates significantly in time and space, necessitating root responses to diverse and changing environmental cues. It has been found that NO_3_^-^ supply enhances NH_4_^+^ uptake in rice (Zhao et al., 2008). Therefore, a deeper understanding of the interaction between NO_3_^-^ and NH_4_^+^, and their roles in physiological and biochemical regulation of N uptake, is crucial for improving NUE. In summary, a systematic understanding of the dynamic interactions between genetic regulatory networks and management practices will provide a theoretical foundation and practical guidance for developing N management strategies that are both high-yielding and environmentally sustainable under variable environmental conditions.

### 5.5 Integrative strategies for future NUE improvement

In summary, we have outlined the genetic regulatory factors involved in the transport of NO_3_^-^ and NH_4_^+^, which contribute to efficient N absorption and translocation. We further discussed the key genes regulating NUE in rice, highlighting their potential to significantly improve both crop yield and NUE. Although substantial progress has been made in understanding the genetic architecture and molecular mechanisms underlying NUE in rice, there remain significant gaps in our knowledge of the complex genetic networks governing NUE regulation. This calls for an in-depth exploration of the genes and regulatory elements affecting NUE through advanced genomic technologies and bioinformatics tools. Future studies should focus on elucidating the functions of these genes, their interactions, and their responses to nitrogen availability under varying environmental conditions. Ultimately, by leveraging strategies such as multi-gene pyramiding, in-depth analysis of signaling regulatory networks, and the mining of elite genetic resources, it is expected that rice NUE can be further improved, facilitating the development of high-yield, environmentally sustainable rice varieties.
